# SMADS-Mediate Molecular Mechanisms in Sjögren’s Syndrome

**DOI:** 10.3390/ijms22063203

**Published:** 2021-03-21

**Authors:** Margherita Sisto, Domenico Ribatti, Sabrina Lisi

**Affiliations:** Department of Basic Medical Sciences, Neurosciences and Sensory Organs (SMBNOS), Section of Human Anatomy and Histology, University of Bari “Aldo Moro”, I-70124 Bari, Italy; domenico.ribatti@uniba.it (D.R.); sabrina.lisi@uniba.it (S.L.)

**Keywords:** SMAD, Sjögren’s syndrome, epithelial-mesenchymal transition, fibrosis, TGF-β, inflammation

## Abstract

There is considerable interest in delineating the molecular mechanisms of action of transforming growth factor-β (TGF-β), considered as central player in a plethora of human conditions, including cancer, fibrosis and autoimmune disease. TGF-β elicits its biological effects through membrane bound serine/threonine kinase receptors which transmit their signals via downstream signalling molecules, SMADs, which regulate the transcription of target genes in collaboration with various co-activators and co-repressors. Until now, therapeutic strategy for primary Sjögren’s syndrome (pSS) has been focused on inflammation, but, recently, the involvement of TGF-β/SMADs signalling has been demonstrated in pSS salivary glands (SGs) as mediator of the epithelial-mesenchymal transition (EMT) activation. Although EMT seems to cause pSS SG fibrosis, TGF-β family members have ambiguous effects on the function of pSS SGs. Based on these premises, this review highlights recent advances in unravelling the molecular basis for the multi-faceted functions of TGF-β in pSS that are dictated by orchestrations of SMADs, and describe TGF-β/SMADs value as both disease markers and/or therapeutic target for pSS.

## 1. Introduction

### 1.1. Fibrosis in Sjögren’s Syndrome

Salivary gland (SG) fibrosis is a recently associated feature of Sjögren’s syndrome (SS), a chronic inflammatory autoimmune disease and multisystem exocrinopathy characterised by dry mouth and dry eyes with wide-ranging extra-glandular involvement [1]. As can be deduced from daily clinical practice, an acute inflammation can be recovered quickly, but chronic inflammation characterizing SS, will progress to irreversible fibrosis with SGs damage and impaired regenerative capacity. It gives rise to accumulation of excessive connective tissues and extracellular matrix (ECM) components, eventually resulting in SG dysfunction [2]. Until recently, researchers have been focusing on studying the mechanisms of inflammation-mediated SG fibrosis. Fibrosis is a common consequence of tissue damage and inflammation [3] and several diseases genetically related to SS have well-described fibrotic components, including primary biliary cirrhosis, systemic sclerosis, ulcerative colitis, and systemic lupus erythematosus [4,5]. Actually, SG fibrosis has been accepted as SS feature [6]; in fact, a positive relationship between biopsy focus score and SG fibrosis, and between ocular surface damage and fibrosis were detected [7]. Chronic inflammation seems to be the principal actor in SG fibrosis in which CD4+ T cells, macrophages and epithelial cells all play roles in pathological accumulation of ECM components and are commonly found in glandular lesions in SS [8,9].

### 1.2. Epithelial-Mesenchymal Transition (EMT) and Fibrosis

Recently, the role of epithelial cells in SG fibrosis has received great attention due to the understanding of the mechanisms underlying a process called epithelial-mesenchymal transition (EMT). EMT, a system whereby differentiated epithelial cells undergo transformation into a mesenchymal phenotype giving rise to fibroblasts and myofibroblasts, is increasingly recognized as playing an integral role in the process of repair and scar formation following epithelial failure in several tissues [2]; furthermore, EMT has recently been recognized as a process also involved in the onset and progression of fibrotic phenomena [10,11]. The knowledge of the molecular mechanisms underlying EMT have demonstrated that the fully differentiated epithelial cells, with a state considered immutable, can undergo significant changes in morphology, function, and polarity [10]. These studies have led researchers to believe that differentiated cell type, such as a tubular or acinar SG epithelial cell (SGEC), with a wide set of characteristic secretory, transport, and structural functions, could radically change its transcriptional process transcribing genes characteristic of mesenchymal cell type [12]. The implications of these findings were very important and the recent explosion of knowledge in the biology of cellular differentiation has highlighted, for example, the implication of the EMT process activation in oncogenic transformation, where the loss of epithelial characteristics and subsequent acquisition of mesenchymal features is a critical program to epithelium-derived cancers progression and invasion [13,14]. More recently, the capacity of various epithelial cells, including SGEC, to undergo dramatic changes to give rise to a mesenchymal phenotype has been interestingly linked to a condition of chronic inflammation [15,16,17,18]. In addition to the study of EMT, recently, several investigations focused on endothelial to mesenchymal transition (EndMT); in cerebral cavernous malformations (CCM), also commonly known as cavernous hemangiomas or cavernomas, which represent common cerebral vascular malformations, Maddaluno et al., showed that endothelial-specific disruption of the CCM1 gene in mice induces EndMT [19], which contributes to the development of vascular malformations such as vascular dysplasia and fragility in CCM [20,21,22]. EndMT in CCM1-ablated endothelial cells is mediated by the up-regulation of Transforming growth factor-β (TGF-β) and bone morphogenetic protein (BMP) signalling, and the consequent EndMT activation results crucial in the onset and progression of CCM disease [19]. In this context, a recent study highlighted a role for EndMT in brain arteriovenous malformation disease (AVM), a congenital defect affecting brain microvasculature, demonstrating that germline mutations in several genes related to TGF-β/BMP signalling are found to contribute to EndMT in AVM [23,24].

### 1.3. TGF-β, Master Regulator of EMT-Dependent Fibrosis

TGF-β has a critical role in cellular responses, such as development, proliferation and differentiation. TGF-β is, also, the master regulator to drive EMT-dependent fibrosis in several organs including the SGs [3,25,26]. EMT in response to TGF-β1, during fibrotic process, is activated predominantly through the activation of SMAD-mediated pathways, although non-SMAD signalling has also been implicated in response to a broad range of context-dependent cellular circumstances [27]. The trigger of the TGF-β family receptors mediates an intracellular signalling cascade that culminates in phosphorylation-dependent SMADS transcriptional activation [28]. Based on these data, in this review we will summarize the major advancements that have been made in our understanding of the roles that SMAD family proteins play in the mechanisms underlying TGF-β-mediated EMT in SS. Comprehension of the downstream mediators which regulate the TGF-β/EMT signalling pathway will stimulate further consideration of the cellular mechanisms of fibrogenesis in the SS SGs, potentially leading to innovative avenues of investigation and treatment of SGs dysfunction.

## 2. TGF-β Signal Transduction

TGF-β exists in the mammalian under three small, secreted signalling isoforms TGF-β1, TGF-β2 and TGF-β3, that are potent regulator of cells growth, differentiation and immune cellular function [29,30]. Among these three isoforms, TGF-β1 is generally considered to be the major or predominant isoform, predominantly expressed in the immune system and is believed to be an important pleiotropic and immunoregulatory cytokine [29,30]. TGF-β1 exerts powerful pro- or anti-inflammatory functions depending on the pathological context because TGF-β1 can contribute to the differentiation of both regulatory T cells and inflammatory Th17 cells; furthermore; TGF-β1 might be, also, down-regulated in some autoimmune diseases, and, for example, mice deficient in TGF-β1 develop a multiorgan autoimmune inflammatory disease leading to death in a short time [31] and various transgenic mice whose T cells are unresponsive to TGF-β1 develop autoimmune diseases [31,32]; on the other hand, TGF-β1 is overproduced in many pathological conditions, including those characterized by a fibrotic evolution such as pulmonary fibrosis, glomerulosclerosis, renal interstitial fibrosis, cirrhosis, Crohn’s disease (CD), cardiomyopathy, scleroderma and chronic graft-vs-host disease [3,33]. Regarding the non-canonical SMADs-independent TGF-β signals, they are generally activated by the interaction of ligands that do not belong to the TGF-β family to tyrosine kinase receptors Unlike other cytokines, TGF-β1 is synthesized in an inactive complex with its pro-domain, which represents the pre-pro-TGF-β1 precursor. This precursor is modified intracellularly prior to secretion through the cleavage of the C-terminal pro-region from the N-terminal portion of the protein. The C-terminal pro-region is referred to as the latency-associated peptide (LAP) while the N-terminal region is called the mature TGF- β1 or active TGF- β1. Then, the precursor dimeric protein LAP/TGF-β binds to the latent TGF-β-binding protein [LTBP], and the LTBP/LAP/TGF-β complex is then secreted from cells and bound to collagen and other ECM proteins [34,35]. At this stage, the complex cannot interact with its receptor and has no biological effect. The mechanism of activation of LTBP/LAP/TGF-β1 complex may be varied and context-dependent and several stimuli, such as low pH, proteolysis, and binding to the cell surface proteins have been identified as capable to liberate active TGF-β1 [35,36]. When TGF binds to its receptor (TGF-βR), the activated main pathway derived from a complex set of interactions between regulators, activating factors and inhibitors [37,38,39]. The binding of TGF-β to the TGF-βR primarily induces SMAD transcription factors, represented consequentially by two receptor-associated SMADs, SMAD2 and SMAD3 that are directly phosphorylated and activated by TGF-βR, followed by the activation of a common SMAD, SMAD4 [40,41,42]. The activated SMAD-complex translocate into the nucleus, where they interact in a cooperative manner with sequence-specific DNA-binding cofactors and transcriptional coactivators or corepressors to regulate the transcription of target genes. In this context of canonical SMAD-mediated TGF-β signalling mechanisms, the activated receptors also signal through other molecular transducers, represented, for example, by the mitogen-activated protein kinase (MAPK) pathways, including the extracellular signal-regulated kinases (Erks), c-Jun amino terminal kinase (JNK), p38 MAPK, as well as the IκB kinase (IKK), phosphatidylinositol-3 kinase (PI3K) and Akt, and Rho family GTPases, which are collectively known as alternative non-canonical SMAD-independent pathways [43,44]. Therefore, TGF-β exerts its regulation of target cell function via a range of mechanisms. Both SMAD-dependent and SMAD-independent signalling pathways are tightly tuned to induce a cascade of events that results cell-type-specific or context-dependent and which has the ability to cross talk with various other signalling pathways.

## 3. SMAD Proteins and Their Role in Signal Transduction

**Figure 1:**
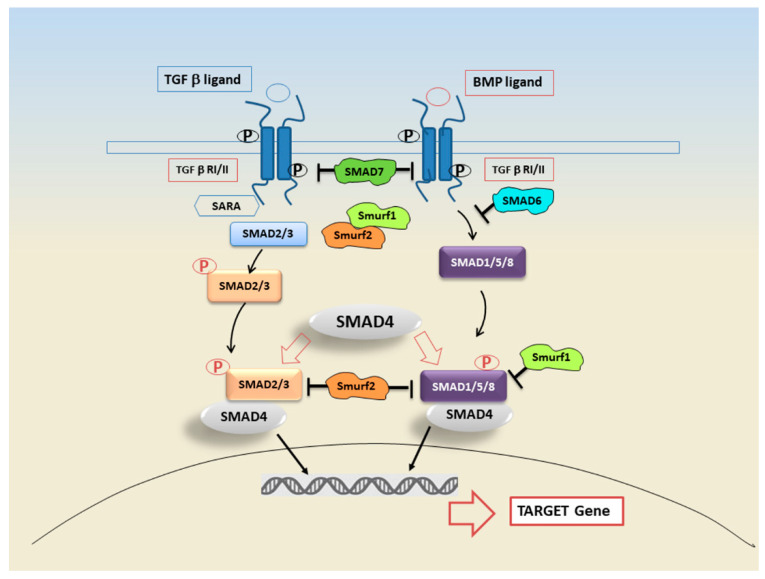
TGF-β signalling is triggered through BMP or TGF-β ligands. Ligands bind the TGF-β receptor II (TGF-βR-II) which recruits and phosphorylates TGF-β receptor I (TGF-βR-I). In the TGF-β signalling pathway, TGF-βR-I phosphorylates the SMAD2 and 3, or SMAD1, 5, and 8. The cofactor SMAD4 forms a heterotrimeric complex with SMAD2/3 and SMAD1/5/8. Activated SMAD complex, translocate into the nucleus interacting with transcription factors and modulating or repressing the gene transcription. Feedback regulation is mediated by inhibitory SMAD 6/7. Both SMAD6 and SMAD7 are in turn induced by TGF-β receptors and regulated through the help of SMAD ubiquitination regulatory factors (Smurfs) 1 and 2.

SMAD proteins represented specific substrates for type I receptor kinases, known to be able to trigger a signalling cascade [40]. Proteins belonging to the SMAD family were first identified in the fruit fly Drosophila melanogaster by Sekelsky et al. who found that an intracellular protein named Mad (mothers against decapentaplegic) mediates the signalling of decapentaplegic (dpp), a member of the TGF-β superfamily corresponding to mammalian bone morphogenetic proteins (BMPs) 2 or 4 (BMP-2/4) [45,46,47]. The discovery of orthologous proteins in *Caenorhabditis elegans* (Sma-proteins) led to the combination of the two names in SMAD [48]. SMADs act as regulators of BMPs, members of the TGF-beta superfamily [49,50]. In accordance to current knowledge, the SMADs are the only TGF-β receptor substrates and TGF-β-downstream effector with a demonstrated ability to propagate a transcriptional signal. The mechanism of activation of the TGF-β receptors has been sufficiently clarified for some decades [38]. Briefly, signalling is initiated by binding the growth factor to a specific pair of transmembrane protein serine/threonine kinases, known as Receptor types I and II; following this event, the phosphorylation and activation of the Receptor type I, which until that time was catalytically inactive, occurs by the other kinase Receptor type II [51]. The activated type I receptor phosphorylates the subgroup of SMADs, known as receptor-regulated SMADs (R-SMADs), which then move into the nucleus [38,52,53]. SMADs are expressed ubiquitously throughout development in practically all adult tissues [54,55,56], and many of them (SMAD2, SMAD4, SMAD5, SMAD6 and SMAD8) derived from alternatively spliced mRNAs [54]. Functionally, SMADs fall into three subfamilies (Figure 1): the first comprises the above cited R-SMADs (SMAD1, SMAD2, SMAD3, SMAD5, SMAD8), which become phosphorylated by the type I receptors; in particular, all R-SMADs share an amino-terminal Mad homology 1 (MH1) domain, a central proline-rich linker, and a carboxy-terminal MH2 domain. Their activation is dependent on the type I receptors that phosphorylate a common Ser-X-Ser motif present at the extreme carboxyl terminus of the MH2 domain [57,58]; the second subfamily is represented by the common mediator SMADs (Co-SMADs: SMAD4); practically, the activation of the R-SMADs is continued by their assembly with the co-SMAD, SMAD4, leading to the complex translocation to the nucleus for SMAD-target genes transcription [59]. Finally, into the third subfamily fall inhibitory SMADs (I-SMADs: SMAD6 and SMAD7), which antagonize the TGF-β signalling pathway. I-SMADs structure lacks an MH1 domain and the SSXS motif, but retain a conserved MH2 domain. Recent discoveries clarified that I-SMADs antagonize the SMAD signalling pathway by associating with the type I receptor, recruiting Smurf1 or Smurf2 E3 ubiquitin ligases, and also through binding the receptor-phosphorylated R-SMADs and interfering with the association with co-SMAD, or interacting with DNA and nuclear SMAD complexes [38,52,60] (Figure 1).

Recently, ubiquitin-dependent mechanisms have emerged as essential regulatory elements controlling cellular levels of SMADs and TGF-β-dependent EMT. The HECT (homologous to E6AP C-terminus domain) E3 ubiquitin ligase known as WWP2, together with two WWP2 isoforms (N-terminal, WWP2-N; C-terminal WWP2-C), were identified as novel SMAD-binding partners. These isoforms interact with SMAD2, SMAD3 and SMAD7 in the TGF-β signalling pathway, modulating the inhibition or activation of SMADs-mediated cascade [61,62,63].

## 4. SMAD and Non-SMAD Pathways in TGF-β Signalling

The physiological responses to TGF-β activation are diverse and vary amongst different cell types and environmental conditions. The TGF-β1/SMAD/Snail pathway is a particularly dynamic system leading to the TGF-β-stimulated EMT, that induces the fibrotic process linking to EMT program in several diseases [44,64,65,66].

**Figure 2:**
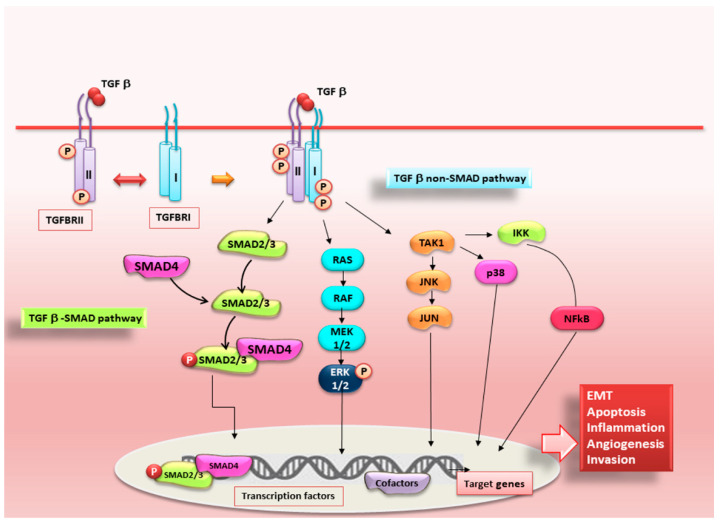
Schematic illustration of canonical and non-canonical TGF-β signalling pathways. Canonical SMAD-dependent TGF-β signalling is initiated by TGF-β ligand binding to receptors TGFBRI/RII, which once activated, lead to the phosphorylation and activation of SMAD2/3 which, in turn, binds to cofactor SMAD4. The trimeric SMAD complex translocate into the nucleus, where they interact with other transcription factors to regulate target gene expression. In the SMAD-independent pathways, the TGF-β receptor complex transmits its signal through other factors, such as RAS-RAF-MEK-ERK. Once activated, ERK1 and ERK2 can facilitate EMT by increasing the expression of EMT transcription factors. Moreover, activated TAK1/JNK/JUN factors act to regulate cellular apoptosis and proliferation, whereas they can also mediate metastasis, angiogenesis and cellular growth through other transcription factors.

All three forms of TGF-β, once bioavailable, interact with the surface of the target cell using the same receptors: type I (RI, or ALK5); type II (RII) and type III (RIII, or betaglycan) [67]. TGF-β binds a homodimer of TGF-β type II receptors (TβRII) and the TGF-β-TβRII complex providing a structural interface that facilitates stable complex formation with a homodimer of the TGF-β type I [68]. Once that the receptor and scaffold proteins are activated, this complex leads to the phosphorylation of regulatory SMAD2/3 proteins to form the Receptor-mediated-SMAD (R-SMAD)/co-regulatory SMAD4 complex [69]. In unstimulated cells, SMADs constitutively shuttle between the cytoplasm and nucleus. Upon ligand stimulation, this SMAD complex accumulates in the nucleus where, interacts with the general transcription machinery to modulate the expression of 300 target genes [70]. The transcription of the genes encoding the zinc finger transcription factor Snail, for example, was successfully activated [71,72]. Snail plays an active role in driving the EMT system which affects the loss of epithelial markers as E-cadherin and claudins and, contemporary, the upregulation of mesenchymal markers such as vimentin and fibronectin [73]. Peinado *et al*., demonstrated, in an experimental model represented by Madin-Darby canine kidney cells treated with TGF-β1, that Snail acts as a strong repressor of the expression of E-cadherin. In these cells, a TGF-β1-dependent EMT program was activated, suggesting that Snail is involved directly in the TGF-β1 signalling [74]. Functional studies have showed that SMADs have an essential role in TGF-β-EMT-dependent fibrosis. In particular, SMAD3 gene silencing inhibits EMT process activation in response to TGF-β stimulation or mechanical stretch in renal tubular epithelial cells [75], and causes a reduction of the migratory ability of these cells in response to TGF-β [76]. Conversely, SMAD2 may play an antagonistic role in the EMT program in vivo. Therefore, interestingly, there is a complex collaboration between SMAD2 and SMAD3, where cooperation and antagonism occur simultaneously in the same cellular context. In addition, studies in the human skin cancer patients demonstrated that the loss of the SMAD2 was linked with trans-differentiation, and with the strong reduction of E-cadherin expression [77]. Moreover, in human skin cancer patients frequently an under expression of SMAD2 was observed. Among EMT-linked target genes, overexpression of the Snail appears to be associated with SMAD2 loss-triggered EMT [78]. There is now ample evidence that suggests SMAD2 could either compete with or impede the capacity of SMAD4 to bind SMAD3 and induce the Snail promoter. Therefore, the loss of SMAD2 determines an intensive binding capacity of the SMAD3/4 complex to the promoter of the Snail target gene, thus provoking the increase of the progression of EMT [77]. Likewise, Ju et al. demonstrated that SMAD2 deletion in the hepatocytes derived from knockout mice underwent the induction of the EMT program, while SMAD3–/– hepatocytes preserved all their epithelial characteristics and did not exhibit molecular alterations of the EMT system [78]. Like that of SMAD3, the role of SMAD4 is indispensable for the activation of EMT. SMAD4 gene knockdown, using RNA interference strategy or transfection with a dominant negative mutant form of SMAD4, resulted in retained E-cadherin expression in a pancreatic cancer cell line [79]; in association with the abolition of type I collagen synthesis in vitro, and with strong diminished bone metastasis in vivo [80]. The same observations were made in a study conducted on adenocarcinoma, reporting that genetic ablation of SMAD4 determines the conservation of epithelial markers and inhibition of the EMT [81]. TGF-β family signalling is finely modulated through a variety of positive and negative regulators, identified recently [68]. For example, the I-SMADs, SMAD6 and SMAD7, are included in the list of the inhibitors, since they act as negative regulators of the TGF-β signalling triggered by EMT [82,83,84,85]. Particularly, SMAD7 inhibits the EMT activation induced by TGF-β in multiple tissues [86,87]. Nevertheless, SMAD7 is tightly associated to the activation of TGF-β in the case of various diseases with chronic inflammatory features, which often induce EMT-dependent fibrosis process. It was recently demonstrated that SMAD7 is overexpressed in the colonic mucosa when chronically inflamed, and in precancerous conditions [88]. This finding was confirmed by inhibiting SMAD7 through the oral administration of SMAD7 antisense oligonucleotide in vivo, leading to diminished inflammatory state in mice affected by colitis [88]. SMAD7 gene silencing, indeed, restored TGF-β1 activity, thus blocking inflammatory cytokines release and resolving the colitis in mice [88]. Similarly, oral administration of anti-SMAD7 antisense oligonucleotide to patients with active CD determines clinical symptoms remission [89]. Regarding the non-canonical SMADs-independent TGF-β signals, they are generally activated by the interaction with tyrosine kinase receptors of ligands that do not belong to the TGF-β family of tyrosine kinase receptors [89]. The activated TGF-β1 receptors trigger an intensive response through a wide range of signal transducers [90]; the active role of these non-SMAD pathways in TGF-β1/EMT-dependent fibrosis was recently showed utilizing chemical inhibitors directed against one or more of these pathways. The PI3K/Akt pathway is involved, even in EMT, in TGF-β-mediated fibroblast proliferation and morphological change characteristic of the EMT program. The use of imatinib mesylate, a chemical inhibitor, it was demonstrated to prevent TGF-β1/EMT-dependent fibrosis in the lung [91]. Furthermore, using specific small-molecule inhibitors that inhibit the MAP kinase or PI3 kinase pathways, Janda et al. evidenced that the Raf-MAP kinase pathway as synergistically intricate in TGF-β signalling, provoking the progression of tumour events and metastasis in EpH4 mammary epithelial cells through EMT activation [92]. A representative scheme of SMADs-canonical and SMADs- independent pathways is represented in Figure 2.

Based on considerable scientific progress the inhibition of the EMT through the identification of TGF-β antagonists that block both SMAD- and non-SMAD-dependent pathways may be among the most important priority research goal for the prevention of cancer and chronic inflammatory diseases progression. Potential strategies interfering with TGF-β ctivity for fibrosis therapy are summarized in Table 1 [93].

## 5. SMADs Signalling Pathways Activated by TGF-β1-Dependent EMT in pSS

**Figure 3:**
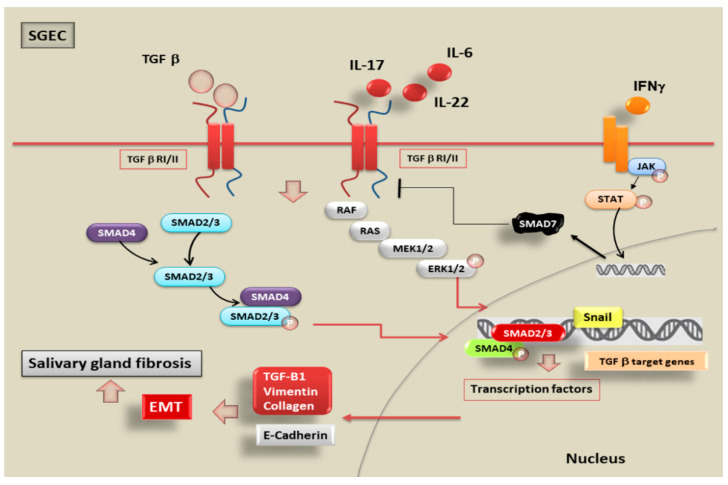
Hypothetical scheme illustrating TGF-β/EMT signalling in SS. TGF-β activates both the canonical SMAD2/3 pathway and the non-canonical MAPK pathway, triggering the EMT process. An inflammatory microenvironment including proinflammatory cytokines such as IL-17, IL-22, and IL-6 may induce EMT through the TGF-β/SMAD and non-SMAD signalling pathways. The activation of transcription factors such as Snail, promotes the prolonged activation of EMT, repressing epithelial marker genes, as E-Cadherin, and activating genes regulating the mesenchymal phenotype, as Vimentin and Collagen Type 1. The epithelial cells trans-differentiated in myofibroblasts are responsible for progressive SG fibrosis. Alternatively, TGF-β can trigger the signals via non-SMAD pathways, such as the MAPK signalling cascade including phosphorylated ERK1/2, that ultimately lead to the activation of the EMT-dependent fibrosis program. An alternative hypothetical mechanism was also showed mediated by IFN-γ that, through the Jak1/Stat1 activation, causes the SMAD7-mediated inhibition of TGF-β signalling.

Recently, considerable attention has been paid to the chronic inflammatory disorders of pSS, and although inflammatory status is often associated with pathological fibrosis, the mechanisms that lead to this condition and the key role of EMT in linking inflammation and SG fibrosis are not clear. Emerging evidences suggest that epithelial cells are also an important source of myofibroblasts in organ fibrosis [94,95] and this trans-differentiation is evaluated as a tightly specialized system of the EMT process that may be a central event in the SG fibrosis. Therefore, the chronic inflammatory microenvironment common to fibrotic and inflammatory disease has emerged as a decisive element in triggering the pathological EMT program [94]. Despite the growing interest of the EMT in several organs, the precise molecular mechanisms through which EMT occurs in SGEC playing a role in the induction of the SG fibrosis in pSS are still poorly understood. Recently, to clarify these doubts, Sisto and collaborators have applied different approaches to investigate EMT process in SGs [26]. Findings obtained demonstrated that in SG tissues, derived from biopsies of patients affected by pSS, the levels of TGF-β1 protein are increased in comparison to those found in healthy SGs tissue, and, since the signal triggered by TGF-β1 is involved during the fibrotic condition, the authors analysed whether the TGF-β1 signalling pathway is activated in human pSS SGs [96]. As expected, this investigation demonstrated that TGF-β1 activates the members of SMAD family through SMAD2/3 phosphorylation; in addition, the expression of the co-SMAD protein, SMAD4 is markedly elevated in the pSS tissue compared with healthy SG tissues. Interestingly, recent evidences revealed a strong positivity for EMT mesenchymal phenotype factors as vimentin and collagen type I in pSS specimens; furthermore, Snail, a transcriptional repressor and promoter of EMT, is over-expressed in comparison with normal SG tissue. On the other hand, the expression levels of epithelial marker E-cadherin was decreased in diseased SG biopsies, evidencing that TGF-β1 triggers the EMT program in SGEC through the canonical TGF-β1/SMAD/Snail signalling [26,97]. Results obtained at the molecular level were confirmed by microscopic investigations carried out in vitro, clearly evidencing phenotypic changes characteristic of EMT in SGEC, in response to TGF-β1 treatment; epithelial cells have undergone phenotypic transition into myofibroblasts, the key effector cells of the fibrotic states [97,98]. Interestingly, several lines of findings highlight that SGEC derived from healthy SG biopsies, exposed to TGF-β1 stimulation, acquired a more fibroblast-like morphology; whereas when cultured in the absence of TGF-β1 or in the presence of the TGF-β1-pathway inhibitor SB-431542, the cells conserved a classic cobblestone epithelial morphology [64]. In this context, a recent study, compared the gene and protein levels of SMAD2/3/4, Snail, E-cadherin, vimentin, and collagen type I isolated from TGF-β1-treated SGEC and the expression levels of the same factors in pSS SGEC. An interesting finding was obtained through this approach, reporting that both regulatory-SMAD 2, 3, and co-SMAD 4 and Snail factor is highly expressed both at gene and protein level following TGF-β1 treatment of healthy SGEC reaching values comparable to those observed in pSS SGEC. In addition, in the cells treated with TGF-β1 as well as in pSS SGEC, an important reduction in the epithelial phenotype marker E-cadherin and an increase in the mesenchymal phenotype markers, vimentin and collagen type I, were demonstrated. All these evidences confirmed that TGF-β1 induces the EMT via the TGF-β1/SMAD/Snail canonical signalling pathway [65,97], so modulating and promoting the progression of the EMT-dependent fibrosis in pSS SGEC (Figure 3).

## 6. Inflammatory Mediators Trigger EMT in pSS through the Activation of TGF-β1/SMADs Canonical and Non-Canonical Pathways

Once the activation of the EMT program has been demonstrated in pSS as dependent on the activation of the cascade mediated by TGF-β1, researchers wondered whether the inflammatory microenvironment could influence the activation of SMADs and mediate EMT-dependent fibrosis. The ability of pro-inflammatory cytokines to directly drive EMT through signalling pathways involved in the pathogenesis of pSS has not until recently been investigated, but innovative data were accumulated providing evidences that inflammatory mediators characterizing pSS play a role in both the initiation and the perpetuation of pathological SG fibrosis [98,99]. Inflammatory cell factors present in injured SG of pSS patients amplified the epithelial-derived cytokine cascade [98,99] and some of these cytokines present in abundance in inflamed SG appear to be directly involved in the activation of the EMT program in pSS by acting on the activation of SMADs proteins. Interestingly, considerable levels of interleukin (IL)-22, strictly associated with a reduced salivation, have recently been identified in pSS patients’ sera [100,101]. Additionally, a recent study reported that the pro-inflammatory cytokines IL-22 and IL-17 are markedly present in the SGs of patients affected by pSS, both correlated with the degree of inflammatory status [17]. Furthermore, contemporary expression of IL-17 and IL-22 in the inflamed pSS SG seems to suggest a central key role in the EMT program. As hypothesized, IL-17 and IL-22 were demonstrated to promote the activation of the EMT-dependent fibrotic cascade in healthy SGEC and, furthermore, a combined action of both IL-17 and IL-22 accentuates the progression of the EMT system [17]. To demonstrate the role of SMADs in this pro-inflammatory cytokines-mediated EMT-dependent fibrosis program activated in pSS, the investigations have currently focused on IL-17 because accumulated data support a pivotal role of IL-17 as an attractive target for the individuation of new treatment for autoimmune diseases [102,103]. Recent findings suggested an unexpected role for IL-17 which seems to activate both the SMAD-mediated canonical pathway and the non-canonical SMAD independent pathway in pSS. In particular, the induction of Erk 1/2 signalling by TGF-β1, in healthy SGEC with IL-17 as stimulus was demonstrated independently from the concomitant activation of the canonical IL-17-dependent TGF-β1/SMAD signalling. Indeed, blocking SMAD2/3 phosphorylation and activation through the use of the specific inhibitor SB431542 the basal or IL-17-TGF-β1-stimulated phosphorylation of Erk1/2 was not involved, showing that both the canonical SMAD2/3 and non-canonical Erk 1/2 pathways are responsible for IL-17-mediated TGF-β1-induced EMT [64]. These observations led to compelling evidence that IL-17 is one of the principal factors inducing EMT in pathological organ fibrosis in chronic inflammatory diseases. Research on the role of pro-inflammatory cytokines in the activation of SMAD proteins during TGF- β1-dependent EMT in pSS is reaching further interesting progress in recent times because experimental in vitro models of pSS demonstrated that IL-6 can also trigger morphological phenotypical changes from epithelial to mesenchymal phenotype, in a dose-dependent manner, in healthy SGEC. These findings, which require further investigation, emphasize the hypothesis that dysregulated expression of IL-6 may promote the EMT-dependent fibrosis, offering a more complete understanding of the role that chronic inflammation characterizing pSS could play in the induction of EMT-dependent fibrosis in SGs [18] (Figure 3).

## 7. Future Perspectives on Alternative Molecular Mechanisms Mediated by SMAD in SS

### 7.1. MicroRNAs and TGF-β/SMAD Signalling in SS

**Figure 4:**
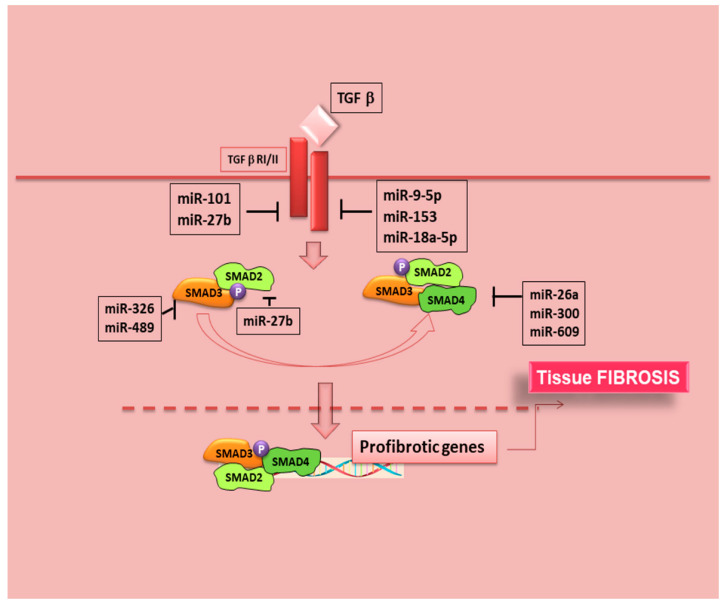
Modulation of the TGF-β signalling pathway by miRNAs. In response to TGF-β signal, the expression of a cohort of miRNAs is modulated. Several miRNAs are involved in the process of EMT-dependent tissue fibrosis by targeting components of the TGF-β signalling pathway.

Recently, microRNAs (miRNAs) were demonstrated to be important regulators of SMAD3-mediated TGF-β/SMAD signalling during fibrosis (Figure 4, Table 2).

Targeting these miRNAs has shown to effectively inhibit fibrosis in the heart, liver, lung, and kidney diseases [104,105,106]. Fortunately, rapidly advancing progresses in this field were made, and there is an increased interest in understanding the roles of SMADs signalling in SS; recent studies, in fact, have expanded our knowledge, creating an association with the expression of microRNA and the pathological molecular pathways activated in SS. Experiments performed on gene expression in SS CD14+ monocytes have provided important findings for the role of pro-inflammatory genes in SS [107]. MiRNAs can negatively modulate gene translation into protein and, interestingly, miRNA expression dysregulation has been documented in a variety of autoimmune diseases, including SS [108,109,110]. However, the vast majority of published research evaluated miRNA expression in mixed cell populations or tissues, and this may confound the further evaluation of specific miRNA-mRNA target relationships. For the pathogenesis of SS there are interesting advances, because a recent finding showed that SMAD4 gene expression undergoes repression in a subpopulation of pSS patients by SS-associated miRNAs [111,112]. Since the SMAD4 protein acts as an essential common mediator for receptor-regulated SMADs to enter the nucleus, the discovery showing that SMAD4 may be targeted by SS-associated miRNAs suggests the activation of a regulating intranuclear gene transcription mechanisms [111] (Figure 4). The progress in the understanding the mechanism for controlling the specificity of miRNAs in SS was accelerated identifying impact of SS-associated miRNAs on TGF-β signaling; in pSS CD14+ monocytes, the relative gene expression of SMAD2, SMAD3, in addition to SMAD4 was evaluated. SMAD2 and SMAD3 gene expression levels are significantly increased in pSS monocytes compared with healthy controls, although SMAD4 gene expression tended to be reduced [111]. Investigating the sequence analyses of miRNA target sites, it was demonstrated that both SMAD2 and SMAD3 3′untranslated regions contain multiple alternative polyadenylation sites upstream of predicted miRNA binding sites; this means that miRNA-directed inhibition of gene transcript levels could fail in this case, while, on the other hand, a significant association for specific miR-300 and miR-609 with reductions in SMAD4 gene expression in pSS patients’ monocytes was confirmed [111]. Obviously, the exact roles of most miRNAs and the underlying mechanisms in pSS have not been yet clarified, requiring further investigation for successful translation to clinical therapies.

### 7.2. SMAD7 Functions as Inhibitor of JAK/STAT Pathway in SS

The blockade of Janus kinase (JAK)/signal transducer and activator of transcription (STAT) signalling may be considered of therapeutic applicability in pSS. It is well known that the use of JAK inhibitors in pSS is promising because suppression of the JAK/STAT pathway improves sicca manifestations [113,114]. Pringle et al. demonstrated, recently, that SG precursor cells react to pro-inflammatory cytokines by proliferation and apparent cell death, presumably by signalling through the JAK/STAT pathway, implying that inhibitors of JAK/STAT signalling may also interfere with the homeostasis of the SG epithelium [115]. The SG epithelium, including its progenitor cells, is characterized by the activation of TGF/SMAD system as pathway salient to the function of SGEC population and to maintain the SGEC homeostasis. It is well known that TGF-β signalling has been implicated in driving development, cellular proliferation, and differentiation [116,117] and recent findings demonstrated that the expression of TGF-β family members is associated with differentiation of SG progenitor cells into acinar cells [118]. Moustakas et al. demonstrated that the activation of the JAK/STAT pathway determines an increase in the activity of SMAD7, known as the ‘anti-SMADs’, so promoting SG precursor cells differentiation; theoretically, blocking JAK/STAT pathway may prevent differentiation of precursor cells in SG acinar cell and eventual saliva production [119]. Interestingly, SMAD7 activation also enables STAT3 activation [120] and, based on these premises one can hazard that blockade of JAK/STAT signalling could have a therapeutic purpose in pSS. In fact, the JAK/STAT pathway is known to be activated by IFN-γ; TGF-β and IFN-γ have opposite effects on diverse cellular functions [121]: TGF-β signals through the initial phosphorylation and activation of the transcription factors SMADs 2 and 3 [42], whereas IFN-γ primarily activates STAT1 through the Jak1-mediated tyrosine phosphorylation [122]. Recent results indicate that IFN-γ specifically inhibits an early step in the TGF-β-induced activation of SMAD3, using as mediator SMAD7 that acts negatively regulating TGF-β signalling [121,123]. SMAD7 binds to the TFG-β-receptor complex, preventing its interaction with, and phosphorylation of SMADs [124], so trans modulating the TGF-β/SMAD signalling by the IFN-γ/STAT pathway. As supposed, IFN-γ, through the Jak1/Stat1 pathway, determines a rapid increase of the SMAD7 expression, causing the inhibition of TGF-β-mediated SMAD3 phosphorylation and blocking the transmission of the TGF-β signalling to the nucleus (Figure 3) [115]. This mechanism may normally function to balance the activity of these two pathways to regulate SGs physiological functioning, but, could represent a promising mechanism useful for the development of new therapies for the SS disease.

### 7.3. Potential Correlation of BMP6 with SMADs Phosphorylation in SS

TGF-β cytokine family includes, among its members, BMP (Figure 1). BMP expression is increased in minor SGs in SS patients; this overexpression is linked to a SGs hypofunction and to an increased lymphocytic infiltration [125]; in addition, in BMP-6–expressing mice, extensive changes in the ECM composition were observed, that could be related to the fibrosis detected in pSS SGs [126]. Confirming these observations, the overexpression of BMP6 in the SGs of genetic modified C57BL/6 mice results in a SS-like phenotype [126], although the underlying mechanism is not clear. Xu et al. assumed that BMP6 could indirectly determine immune cell infiltration of SGs through the impairment of mesenchymal stem cell function [127]. Other studies suggested that BMP6-induced SG dysfunction was rather associated with an altered glandular cell water permeability or ECM protein modulation [125,126,128]. One of the mechanisms investigated to evaluate the alteration of cell permeability in SGs was based on the study of the expression of aquaporin 5 (AQP5); in this case BMP-6 could act interfering with AQP5 post-translational modification necessary for function, but this hypothesis will require further study [129]. With regards to the role of BMP6 in the activation of TGF-β signalling cascade, recently, using mouse models of SS, it was demonstrated that the inhibition of BMP6 signalling reduced phosphorylation of regulatory SMAD1/5/8 in the SGs leading to a recovery of SG function and to a decrease in inflammatory factors, confirming the role of BMP6 as TGF-β1 antagonist [125]. But, on the other hand, there are experimental evidences that an increased expression of pro-inflammatory cytokines in SS patients could be correlated to BMP-6 signalling, and eventually to the activation of TGF-β1/SMADs signalling cascade [93]; therefore, more work will be required in the future to confirm the possibility that TGF-β/SMADs cascade in SS could be modulated by BMP-6 expression.

## 8. Conclusions

Clearly, important aspects of the cell biology of the SMAD pathway are yet to be understood and the current advances in research into the TGF-β/SMAD signalling pathway could improve our understanding of the molecular mechanisms of chronic inflammatory autoimmune diseases. Here we summarized studies describing the association of deregulation of TGF-β/SMADs signalling with SS. Compared to other human diseases, the current knowledge of how TGF-β pathways lead to various autoimmune abnormalities is limited and causal links between altered function of SMADs and SS have not been generated. Over the last decade, several studies have explored the efficacy and safety of biologic agents in SS and after the failure of tumour necrosis factor-α inhibitors, there has been a considerable and concerted effort to develop drugs that target different components of the canonical TGF-β signalling pathway. Numerous relevant preclinical studies have individuated intracellular targets for SG fibrosis within the canonical SMAD pathway but have not yet reached clinical trial. Indeed, several TGF-β pathways targeting therapeutic candidates have been developed or are under investigation, yet the majority have not achieved satisfying clinical results. We hope that this article will provide a basis for future research aimed at providing more mechanistic insights into immunological abnormalities stemming from deregulated TGF-β/SMADs signalling; taking a network-wide view on TGF-β/SMADs signal transduction could be essential for the future development of targeted therapies for SS.

## Figures and Tables

**Table 1:** Potential therapy interfering with TGF-β dependent fibrosis.

Strategy [93]	Potential Therapy
Block activation of TGF-β receptors inhibitors	Small molecule inhibitors
Block coactivator recruitment and function	Aptamers (Trx-SARA)
Block ligand production or activity	Isotype-specific neutralizing antibodies
	Soluble TβR1-3 receptors
	Antibodies to avß6 integrin
	Natural TGF-β binding proteins (eg. Decorin)
	Nucleic acid-based (antisense, ribozyme, siRNA)
Inhibition of the SMAD Pathway	Physiologic endogenous inhibitor Smad7
	halofuginone (HT-100)
	SIS3, targets SMAD3 phosphorylation
	Adenovirus vector gene transfer of SMAD7

**Table 2:** MicroRNAs regulate TGF-β/SMAD signalling during fibrosis.

Anti-Fibrotic miRNAs	[104,105,106,107,108,109,110,111,112]
miR-101	TGF-ßRI
miR-9-5p	TGF-ßRII
miR-153	TGF-ßRII
miR-326	TGF-ß1, Smad3
miR-27b	TGF-ß1, Smad2
miR-489	Smad3
miR-26a	Smad4
miR-300	Smad4
miR-609	Smad4
